# Acrylamide Intake in Senior Center Canteens: A Total Exposure Assessment Applying the Duplicate Diet Approach

**DOI:** 10.3390/foods14061073

**Published:** 2025-03-20

**Authors:** Marta Mesias, Lucía González-Mulero, Francisco J. Morales, Cristina Delgado-Andrade

**Affiliations:** Institute of Food Science, Technology and Nutrition (ICTAN-CSIC), José Antonio Novais 6, 28040 Madrid, Spain; mmesias@ictan.csic.es (M.M.); lucgon03@gmail.com (L.G.-M.); fjmorales@ictan.csic.es (F.J.M.)

**Keywords:** dietary intake, acrylamide, dietary exposure, risk assessment, senior center canteen

## Abstract

This study conducted a total acrylamide exposure assessment through the daily diet in two Spanish senior centers using the duplicate diet method. Among foods regulated in Europe, only instant coffee provided at senior center 1 (2831 µg/kg) exceeded the benchmark value of 850 µg/kg. The primary contributors to acrylamide intake were French fries (27.3 µg/serving) and Spanish omelet (21.6 µg/serving), followed by stews, soups, and creams (16.1–5.8 µg/serving). Total acrylamide exposure was estimated under lower bound (LB) and upper bound (UB) scenarios, being 0.36–0.40 and 0.48–0.54 µg/kg bw/day, respectively. In the LB scenario, cereal-based products were the largest contributors (≤90.4%), whereas in the UB scenario, other foods/meals, including stews and vegetal dishes, became the main contributors (≤83.4%). The margin of exposure (MOE) for neoplastic effects ranged between 314 and 474, indicating a potential health risk for consumers. These findings emphasize the need to integrate nutritional and food safety considerations when designing diets for elderly populations.

## 1. Introduction

Over recent decades, the increasing life expectancy and evolving societal structures in regions like Europe and North America have significantly elevated the demand for nursing homes, with populations aged 65 and older growing by over 20% [1]. Factors such as the reduction in available caregivers, particularly due to women’s growing workforce participation, and the complexity of managing chronic diseases have accelerated the shift toward professional elderly care services [2]. The acceptance of nursing homes has also been facilitated by advances in specialized care, offering an enhanced quality of life for residents who face loneliness or loss of autonomy. Today, these facilities have become crucial social and healthcare centers, providing both temporary and long-term solutions for elderly individuals in need of comprehensive support [3,4].

Nursing homes play an essential role in maintaining the nutritional health of their residents, addressing specific dietary needs, such as allergies, swallowing difficulties, nutrient deficiencies, and dietary restrictions. Proper nutrition is indispensable for preserving health, managing chronic conditions, and enhancing overall quality of life in older adults. There are different guidelines for designing menus in nursing homes, emphasizing the efficiency and large-scale preparation of food tailored to elderly residents’ dietary needs and preferences [4,5,6].

Several studies, such as the recent NutriCare study, indicate that nursing home residents often have suboptimal nutritional status [7,8]. Common deficiencies in calcium, magnesium, zinc, vitamin D, vitamin B6, and folate have been reported among residents [4]. For instance, studies have shown that over 60% of elderly residents in long-term care facilities suffer from vitamin D deficiency, while nearly half exhibit inadequate levels of calcium and magnesium, exacerbating risks for osteoporosis and cardiovascular issues. Similarly, adequate protein intake has been linked to improved muscle mass and strength, reducing fall risk in elderly individuals [4]. Paradoxically, despite these deficiencies suggesting malnutrition, elevated rates of overweight and obesity are also observed [3,9]. Given this dual burden of malnutrition and overweight, regularly assessing the nutritional profile of nursing home menus and their impact on residents’ nutritional status has become a standard practice. Regular assessments of nursing home menus are crucial, not only to evaluate their nutritional adequacy but also to address emerging food safety concerns, such as process contaminants, which are less frequently examined. Integrating both nutritional and food safety perspectives is imperative to meet the health challenges of an aging population effectively.

Acrylamide is a harmful compound with carcinogenic and genotoxic properties that develops during high-temperature cooking methods like baking, frying, or roasting. Acrylamide formation is linked to the Maillard reaction between the free amino acid asparagine and carbonyl compounds, its major mechanisms of formation [10]. Animal studies have established a link between acrylamide exposure and increased certain cancer risks across all age groups, highlighting a significant health risk for consumers, particularly for individuals who are exposed to high levels of acrylamide over extended periods. In addition to its carcinogenic potential, acrylamide exposure has been linked to other health problems, such as neurotoxic effects, particularly in sensitive populations like pregnant women and children, where brain function and development could be impaired [11].

Due to its potential toxicity, it is crucial to investigate the intake of thermally processed foods and the resulting dietary exposure to acrylamide. While thermally processed foods are widely consumed by the general population, elderly individuals tend to prefer more traditionally prepared meals. This preference is particularly advantageous in nursing center canteens, where such meals are both efficient to prepare and well-suited for large-scale food production. Evaluating dietary exposure to acrylamide in vulnerable populations, such as nursing home residents, is particularly important.

Numerous studies have evaluated acrylamide exposure using various assessment methods, including probabilistic models, dietary surveys, and reference levels of contaminants in food [12]. While those approaches have limitations, they have been used to assess exposure in both the general population and specific subgroups [13,14,15]. The EFSA recommends the duplicate diet approach to improve exposure assessment, as it allows for the accurate determination of actual dietary intake of contaminants [11]. However, despite this recommendation, studies directly analyzing acrylamide exposure across all food items in a complete diet, while also assessing the associated risk, remain limited, particularly among older populations living in specialized care centers [16].

To address this gap, this study aims to evaluate acrylamide exposure in the daily diets of Spanish nursing home residents using the duplicate diet method. The findings are intended to inform dietary planning and policy adjustments to reduce acrylamide intake, improve food safety standards, and promote healthier nutritional strategies tailored to the elderly population. By estimating associated health risks, it seeks to provide valuable insights into the implications of acrylamide intake in this vulnerable population. The findings are expected to contribute to the development of dietary strategies that integrate nutritional adequacy and food safety, ultimately improving the well-being of elderly individuals dependent on institutional care.

## 2. Materials and Methods

### 2.1. Study Design and Food Samples

Two senior center canteens located in Castilla-La Mancha (Spain) were chosen in this study. These facilities were operated by different food service companies, leading to differences in food handling and preparation methods. The study was conducted from February 2020 to December 2021 and followed a comprehensive protocol for data collection and analysis. Each canteen took part in a two-week trial, during which all foods and meals included in the daily menus from Monday to Friday were gathered using the duplicate diet approach (Table 1A–D). The duplicate diet method was chosen for its ability to accurately represent actual dietary intake by collecting identical portions of all meals consumed. Menus included breakfast, lunch, afternoon snack, and dinner, covering the entire diet of the senior adults. A total of 304 foods and meals were gathered throughout the study period. A code was given to each food sample, and pictures were taken before processing in the laboratory. Following the EFSA FoodEx2 classifications system [17,18], each meal was assigned a standard classification based on its composition and cooking procedure. This systematic approach facilitated precise evaluations of acrylamide exposure. For sample preparation, the edible portion of each meal was separated, weighed, and blended using a hand mixer (Taurus, Vital CM, Lérida, Spain). The homogenized samples were then divided into aliquots and stored at −20 °C until further analysis.

### 2.2. Acrylamide Determination by LC-ESI-MS/MS

Chemicals and reagents used for acrylamide determination were as follows: Acrylamide standard (99%), Carrez-I (potassium hexacyanoferrate (II) trihydrate, 98%), and Carrez-II (zinc acetate dihydrate (>99%) were purchased in Sigma (St. Louis, MO, USA). Acrylamide labeled isotopically with ^13^C_3_ (99%) was obtained from Cambridge Isotope Laboratories (Andover, MA, USA). Hexane, methanol (99.5%) and formic acid (98%) were provided by Panreac (Barcelona, Spain). Deionized water, used for all solutions, was obtained from a Milli-Q Integral 5 water purification system (Millipore, Billerica, MA, USA). Cellulose syringe filters (0.22 and 0.45 µm) were purchased from Análisis Vínicos (Tomelloso, Ciudad Real, Spain), while Oasis-HLB cartridges (30 mg, 1 mL) were supplied by Waters (Milford, MA, USA). All solvents, reagents, and chemicals were of analytical grade.

**Figure 1:**
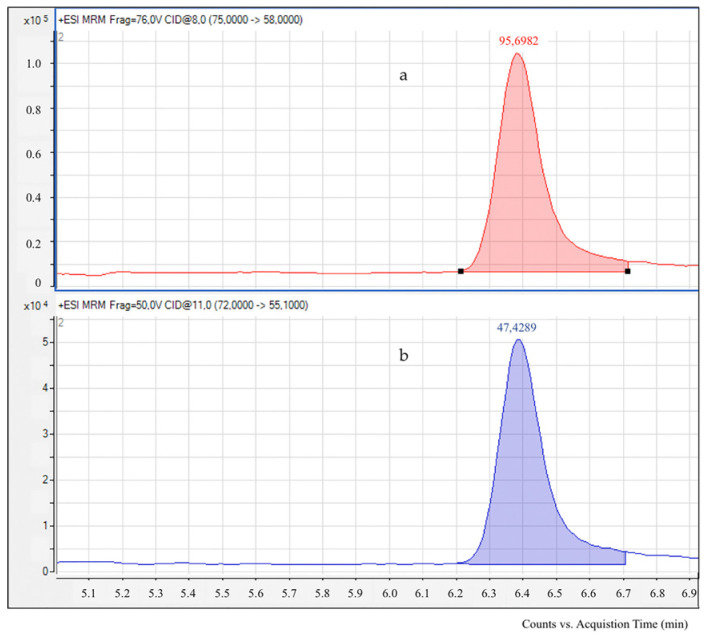
LC chromatograms and mass spectra of acrylamide isotopically labelled with ^13^C_3_ used as an internal standard (**a**) and acrylamide (**b**).

Acrylamide levels in foods and meals were measured using liquid chromatography coupled with electrospray ionization tandem mass spectrometry (LC-ESI-MS/MS), based on the validated method outlined by González-Mulero et al. [19]. An Agilent 1200 liquid chromatograph paired with an Agilent Triple Quadrupole MS detector (Agilent Technologies, Palo Alto, CA, USA) was utilized. Separation was performed on an Inertsil ODS-3 column (250 × 4.6 mm, 5 µm; GL Sciences Inc., Tokyo, Japan) at 30 °C. Isocratic elution was carried out with a mobile phase of formic acid in water (0.2 mL/100 mL) at a flow rate of 0.4 mL/min. The injection volume was 10 µL. Positive ionization mode was employed for electrospray ionization. Under these conditions, acrylamide eluted at 6.4 min. The needle voltage was set to 1.0 kV, with nitrogen as the nebulizer gas at flow rate 12.0 L/min. The temperature of the source was maintained at 350 °C. Signals at *m*/*z* 72.1–*m*/*z* 55.1 were monitored for acrylamide and signals at *m*/*z* 75.1–*m*/*z* 58.1 were monitored for (^13^C_3_)-acrylamide. The fragmentation for the transition *m*/*z* 72.0 > *m*/*z* 55.1 was set at 50 V with a collision energy of 11 V; while for the transition *m*/*z* 75.0 > *m*/*z* 58.1, fragmentation was set at 76 V and collision energy at 8 V. Masses were recorded using multiple reactions monitoring (MRM). For quantitation, the signals at *m*/*z* 75.1 and 78.1 were used, while signals at *m*/*z* 58.1 and 55.1 served for qualification (Figure 1). Matrix effects were considered during quantification by applying the recovery rate of the internal standard in each food or dish analysis. The accuracy, repeatability, and reproducibility of the method were evaluated through the analysis of several samples conducted on the same day (accuracy) by different analysts, which means repeatability, and across multiple days, which means reproducibility. Fortified samples with acrylamide at various concentrations achieved recovery rates ranging from 94% to 103%. Repeatability and reproducibility were tested using different analyses and over multiple days, with relative standard deviations (RSDs) consistently <10%. LOD (limit of detection) LOQ (limit of quantification) were determined by injecting progressively lower acrylamide concentrations and calculating their respective signal-to-noise ratios. The LOD was determined to be 4.5 µg/kg (signal-to-noise ratio of 3), while the LOQ was set at 15 µg/kg (signal-to-noise ratio of 10). The samples were analyzed by duplicate. The results were expressed in µg/kg of the edible portion.

To verify the method’s accuracy, proficiency tests were conducted using the Food Analysis Performance Assessment Scheme (FAPAS). The most recent proficiency test results included test ID number 30117 (coffee, December 2021), test ID number 30118 (crispbread, January 2022), and test ID number 30133 (potato crisps, March 2023), with z-scores of 0.1, −0.1, and −0.2, respectively, demonstrating the reliability of the findings.

### 2.3. Estimation of Acrylamide Exposure

Acrylamide exposure in senior adults consuming food and meals provided in the menus at senior center canteens was estimated by a deterministic approach. The acrylamide contribution from each meal was determined by multiplying the acrylamide concentration in the meal by the weight of its edible portion.

Values for foods with acrylamide levels below the limit of quantification (LOQ, <15 µg/kg) were replaced with zero (lower bound, LB) or the LOQ (upper bound, UB) in accordance with recommendations from food safety studies [11,20]. Dietary acrylamide exposure for senior adults was determined by summing the acrylamide contributions from all foods and meals consumed over one day and normalizing the total by body weight (bw) (Equation (1)). The average daily acrylamide exposure was calculated for centers 1 and 2, considering the single menu option provided each day.

(1) $$\mathrm{Estimated}\;\mathrm{dietary}\;\mathrm{exposure}=\Sigma \frac{\mathrm{acrylamide}\;\mathrm{content}\;\mathrm{of}\;\mathrm{food}\;\left(\frac{µg}{kg}\right)\;\times \;\mathrm{Food}\;\mathrm{consumption}\left(\frac{\mathrm{kg}}{\mathrm{day}}\right)}{\mathrm{Body}\;\mathrm{weight}\;(\mathrm{kg})}$$

Dietary exposure was calculated separately for males and females, using average body weight data for the Spanish population aged 65 years and older: 81 kg for males and 72 kg for females [1]. The results were presented as µg/kg bw/day.

Health risks linked to acrylamide exposure were evaluated by the calculation of the margin of exposure (MOE) for both neurotoxic and neoplastic effects, using Equation (2):

(2) $$\mathrm{MOE}=\frac{BMDL10\left(µg/\mathrm{kg}\;\mathrm{bw}/\mathrm{day}\right)}{\mathrm{Estimated}\;\mathrm{dietary}\;\mathrm{exposure}\;(µg/\mathrm{kg}\;\mathrm{bw}/\mathrm{day})}$$

A benchmark dose lower confidence limit (BMDL_10_) of 430 µg/kg bw/day, obtained from studies in rats, was used to evaluate the risk of neurotoxic effects. MOE values < 125 were considered indicative of possible human health risks for neurotoxicity. For carcinogenic effects, a BMDL_10_ of 170 µg/kg bw/day, based on data from male mice with Harderian gland tumors, was applied. In this case, MOE values < 10,000 were interpreted as indicating a potential risk [11].

### 2.4. Statistical Analysis

Statistical analyses were performed applying Statgraphics Centurion XVI.I software. The results were presented as means and standard deviations (SD). The Shapiro–Wilk test was used to assess the normality of data distribution, and Levene’s test was applied to check for homogeneity of variances. A two-tailed Student’s *t*-test was used to compare acrylamide levels between the two senior centers. A significance threshold of *p* < 0.05 was established for all analyses.

## 3. Results and Discussion

### 3.1. Analysis of Menus Offered in Senior Center Canteens

The analysis of menus provided at two senior center canteens over a two-week trial revealed significant insights into their dietary composition and adherence to nutritional guidelines (Table 1A–D).

Based on AESAN dietary recommendations [6], the menus offered a variety of food groups to ensure balanced, sustainable meals for the elderly. Both centers provided three main meals and two snacks daily, including fruits, vegetables, cereals, dairy, and protein-rich foods. However, there were gaps in fruit (fewer than two servings per day) and legumes, which were below the recommended frequency. Culinary practices aimed to preserve the nutritional value, using methods like boiling, baking, grilling, and frying, of seasonal ingredients for added flavor. Meals were adjusted for dietary restrictions, including low-sodium options and modified textures for residents with swallowing or dental issues. Fortified foods, such as calcium- and vitamin D-enriched dairy products, were also used to address common nutrient deficiencies in the elderly, as highlighted by Sahin and Caferoglu [4].

The guidelines for maintaining balanced meals in Spanish senior centers are outlined in Law 17/2011 on Food Safety and Nutrition [21] and AESAN’s 2022 recommendations [22]. These documents require menus to include a first course, second course, and dessert, using seasonal foods to ensure balanced nutrition. They must also consider seniors’ nutritional status, food preferences, dietary restrictions, and medication interactions that can affect nutrient absorption or cause side effects. Texture is another important factor for those with dental issues, and adequate fluid intake should be ensured, especially through soups and water-rich foods, as well as drinks like milk or herbal infusions at meals.

A recently published document provides updated guidelines for preparing healthy, environmentally sustainable menus in senior centers. It recommends daily servings of 3 vegetables, 2–3 fruits, 3–6 cereals, 3 dairy products, and moderate consumption of potatoes. It also advises a weekly intake of 4 legumes, 3 fish or shellfish, 4–5 eggs, and a limit of 3 servings of meat or meat products [6].

Overall, the menus largely adhered to national dietary guidelines, which emphasizes the integration of sustainable and culturally relevant practices in meal planning for elderly populations. The distribution of food throughout the week was varied, as were the culinary techniques used for cooking. The menus provided at these senior centers conform to the nutritional directives established in international frameworks aimed at fostering healthy dietary practices within these public facilities.

### 3.2. Acrylamide Content in Foods and Meals Provided in Senior Center Canteens

**Figure 2:**
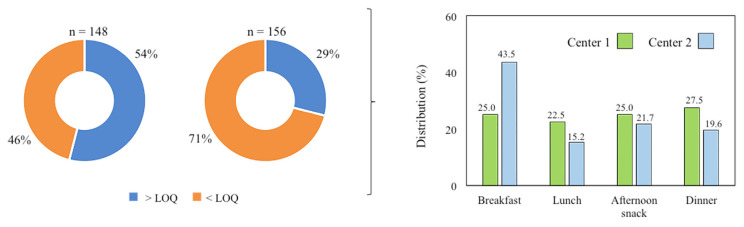
Percentage of foods and meals with acrylamide concentrations exceeding the LOQ and their distribution in the dishes that make up the daily diet in both senior center canteens.

Among the 304 food and meal samples collected from the two senior center canteens, acrylamide was detected in 41% of them. Detailed results are presented in Table 2A,B. At senior center 1, 148 food and meal samples were analyzed, and 54% (80 samples) contained acrylamide levels above the LOQ. The distribution of acrylamide-containing samples was relatively balanced across meal categories: breakfast (25.0%), lunch (22.5%), afternoon snacks (25.0%), and dinner (27.5%) (Figure 2). In contrast, senior center 2 had a lower percentage, with 29% (46 out of 156 samples) above the LOQ. At this center, acrylamide-containing foods were most frequently consumed at breakfast (43.5%), followed by afternoon snacks (21.7%), dinner (19.6%), and lunch (15.2%). This observation suggests that when a diet contains limited acrylamide sources, they are typically found in meals with a higher proportion of industrially processed foods, usually consumed at breakfast or during afternoon snacks.

The highest mean acrylamide concentrations were found in instant coffee and French fries at both centers, along with instant cocoa at senior center 1. For coffee, mean acrylamide levels ranged from 192 µg/kg (center 2) to 2831 µg/kg (center 1), exceeding the benchmark value of 850 µg/kg determined in the European Regulation 2017/2158 [23]. French fries reported mean levels between 213 µg/kg (center 1) and 304 µg/kg (center 2), which remained below the benchmark value of 500 µg/kg. In comparison, previous research has reported acrylamide levels of 187.6 µg/kg in French fries from Colombia [24] and 101.6–1174.8 µg/kg in fries from the Korean market [25]. The results align with previous studies conducted by our research group in public collectivities, which documented high levels of acrylamide in potato crisps and French fries [26,27]. Numerous other studies have also reported elevated acrylamide concentrations in potato-based products such as potato crisps and French fries, compared to other food items [28,29,30,31,32]. Potato-based dishes, such as Spanish omelet (mean 161 µg/kg, Table 2A) and oven-baked potatoes (mean 20 µg/kg, Table 2B), also contributed to acrylamide intake but at comparatively moderate levels.

Similarly, cereal-derived foods ranged from 21 µg/kg in muffins to 122 µg/kg in biscuits, all below the reference values specified by the European Commission for bread (50–100 µg/kg) and biscuits (350 µg/kg) [23]. Previous studies conducted in Hong Kong, Japan, and China identified vegetables cooked at high temperatures as significant dietary sources of acrylamide in their populations [33,34,35]. Vegetable-based preparations exhibited varied concentrations, with roasted red peppers containing 160 µg/kg and sautéed vegetable omelets ranging from 55 to 65 µg/kg. Moisture-rich dishes such as soups and stews generally had low acrylamide levels, but the presence of sautéed components or added spices might explain the detected concentrations. In this regard, mean acrylamide concentrations of 397.0 µg/kg in black pepper and 61.8 µg/kg in white pepper have been previously reported [25]. The remaining foods and meals showed very low acrylamide concentrations, varying between 17 µg/kg, detected in the typical chickpea stew made with pork and beef meat and vegetables, and 40 µg/kg, measured in the fish soup. Both dishes were prepared using boiling methods, which did not promote the Maillard reaction.

### 3.3. Acrylamide Intake in Senior Center Canteens

The estimation of the acrylamide daily intake among elderly residents at the two senior centers was calculated based on the concentrations measured in food samples and average consumption patterns throughout the 10-day study. Calculations were performed for the two exposure scenarios considered: one including only the consumption of foods with acrylamide concentrations above the LOQ (lower exposure scenario; Table 2A,B), and another that additionally incorporated foods with acrylamide levels < LOQ (upper exposure scenario; Table 3A,B).

It was considered that the entire collected edible portion was consumed, so these data reflect the maximum potential acrylamide intake. The acrylamide contribution of each food and meal was determined by factoring the acrylamide concentration in the samples and the size of the edible portion. All portions of food were edible except for the salmon stew with vegetables, rice with pork meat and vegetables, and chicken and garlic stew in the center 1, where the inedible portion was removed, the total portions being 160 g, 319 g and 160 g, respectively. In the center 2, all portions of food were edible except for the fried anchovies, breaded cod, and baked mako shark, the total portions being 47 g, 219 g and 208 g, respectively.

The highest contribution to the acrylamide daily intake among elderly residents was found in potato-based foods, such as French fries (27.3 µg/serving) and Spanish omelet (21.6 µg/serving), followed by stews, soups, and creams, with seafood soup (16.1 µg/serving) and tomato soup (14.0 µg/serving) being the most notable. To estimate the UB scenario, an acrylamide value of 15 µg/kg was set to all dishes and foods with levels below the quantification limit but above the detection limit. With this assumption, among the dishes assigned the LOQ value, the main contributions came from different stews (5.4–7.9 µg/serving) and vegetable-based dishes (below 7.8 µg/serving) (Table 3A,B).

**Figure 3:**
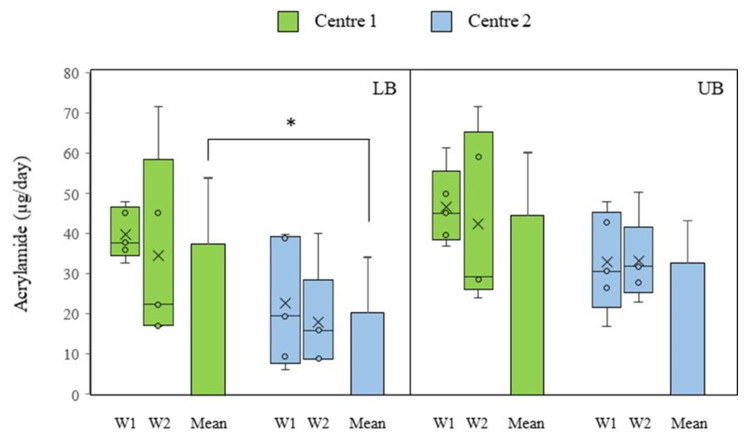
Daily intake of acrylamide in the evaluated senior center canteens. Lower exposure scenario (LB); Higher exposure scenario (UB); Week (W). * The symbol between bars denotes statistical differences between centers (*p* < 0.05).

The variability of the weekly outcomes for each center in both scenarios is depicted through a box-and-whisker plot presented in Figure 3. Furthermore, the figure elucidates the average weekly intake throughout the period of the study. In the LB scenario, daily acrylamide intake ranged from 17.4 to 72.1 µg in senior center 1 and from 6.1 to 40.0 µg in senior center 2. The weekly average for both centers was 37.7 µg/day and 20.5 µg/day, respectively, with statistically significant differences (*p* = 0.021). The overall average for the two canteens was 29.1 µg/day. In the UB scenario, daily intake was considerably higher, reaching an average value of 39.0 µg/day. In this case, daily intake ranged between 24.4 and 72.1 µg in center 1, and between 16.9 and 50.4 µg in center 2, resulting in weekly mean values of 44.8 µg/day and 32.9 µg/day, with no significant differences between them (*p* = 0.068).

Nematollahi et al. [14] reported a mean acrylamide intake of 37.11 µg/day in the Iranian population aged 61–96 years, similar to the estimates from our study. However, a limitation of their study is that it only considered the consumption of foods primarily based on bakery products, various snacks, nuts, and fast food (pizza, hamburgers, and assorted cold cuts). Comparable ranges of acrylamide consumption have been reported in adults from Poland (23.1 µg/day) [36], Japan (4.7–15.8 µg/day) [37], China (14.6 µg/day) [38], the United States (18.1–71.4 µg/day) [39], Italy (33.6 µg/day), and Australia (22.8 µg/day) [40], with even higher values in the European population (261 µg/day) [41].

The relative contribution of food groups to the total daily acrylamide intake was calculated and expressed as a percentage. The results are presented as ranges of contribution (minimum and maximum) for both lower and upper exposure scenarios (Table 4). In the lower exposure scenario, the largest contributors were cereal-based products, accounting for up to 90.4%, followed by other foods/meals (84.6%), potato-based dishes (70.6%), and, to a lesser extent, coffee (33.4%) and cocoa (21.5%). This distribution shifted in the upper exposure scenario, where other foods/meals became the largest contributors (83.4%) followed by potato-based dishes (54.2%), and cereal-based products (43.7%). Coffee and cocoa continued to contribute to a lesser extent, accounting for 23.7 and 15.3%, respectively. The findings were consistent with those reported by other total dietary exposure assessment studies considering coffee, cocoa, cereal-based products, and potato-based dishes [12,25,32,38], some of them also focusing on the elderly population [38]. Similarly, data from the EFSA on elderly individuals highlights these food sources as the primary contributors to the total acrylamide exposure, with other products based on potatoes, cereals, and cocoa accounting for approximately 40% of the total exposure [11]. However, and this is a key aspect that sets our study apart from others, previous investigations typically did not consider other food categories, such as breaded foods, stews, and dishes containing processed vegetables. Although many of these foods contained relatively low levels of acrylamide, their larger serving sizes resulted in a higher contribution to acrylamide intake, making up as much as 80% of the total intake on certain days.

Consistent with the findings of Zhu et al. [35], it is important to consider the variety of foodstuffs that may undergo heat processing under different conditions when modelling dietary exposure to acrylamide. In line with results from total dietary exposure assessment studies conducted on Chinese populations [33,42] and Irish adult populations [5], vegetables contribute to acrylamide exposure. Authors of both investigations also emphasized that only a few studies consider the presence of acrylamide in vegetables other than potatoes. As a result, these foods are often excluded as potential contributors to acrylamide exposure. Our research demonstrates that an important food category contributing to acrylamide exposure—not because of its high contaminant content, but due to its high frequency of consumption and portion size—is being underestimated. From a toxicological standpoint, it is well known that the combined exposure to a toxic substance from multiple small sources can lead to the emergence of unexpected toxic effects over time, even at low levels below the established safe thresholds [43].

### 3.4. Dietary Exposure to Acrylamide in Senior Center Canteens and Risk Assessment

Acrylamide exposure through consumption of the daily diet prepared in senior center canteens, along with the associated risk, was estimated for both the LB and UB scenarios, considering 72 kg as a reference of an average body weight for women and 81 kg for men [1] (Table 5). Daily acrylamide exposure was higher in senior center 1, with significant differences compared to center 2 only in the LB scenario (*p* = 0.021). While differences in the UB scenario were not statistically significant (*p* > 0.05), women exhibited higher exposure levels (0.52–0.62 µg/kg bw/day at center 1 and 0.28–0.46 µg/kg bw/day at center 2) than men (0.46–0.55 µg/kg bw/day at center 1 and 0.25–0.41 µg/kg bw/day at center 2) due to lower average body weight. Consequently, MOE values for both neurological and neoplastic effects were lower for women.

For neurological effects, MOE values across all scenarios exceeded the reference value of 125, meaning no risk. Nevertheless, MOE values for neoplastic effects were significantly lower than 10,000 (reference value), highlighting a considerable health risk. The average MOE values in the LB scenario were 421 for men and 474 for women, decreasing to 314 and 353, respectively, in the UB scenario. These findings underscore the need for targeted measures to reduce acrylamide exposure in elderly institutional diets.

It is also important to consider that acrylamide is only one of many dietary risk factors affecting elderly populations. Overall, the cumulative impact of consuming highly processed foods alongside a low intake of fresh fruits and vegetables may significantly contribute to multiple diet-related diseases. While this study focuses specifically on acrylamide risk assessment, broader dietary improvements could play a crucial role in reducing overall health risks in institutional settings.

The values of exposure observed in this investigation were higher than those observed in other countries. Health Canada [44] reported an average exposure of 0.187 µg/kg bw/day in adults aged 51–70 years, while Wong et al. [33] recorded values of 0.213 µg/kg bw/day in a Hong Kong adult population. Similar lower estimates were observed in Ireland (0.16–0.38 µg/kg bw/day) [5]; the Netherlands (median 0.3 µg/kg bw/day) [45]; France (mean 0.43 µg/kg bw/day) [46]; and China (mean 0.319 µg/kg bw/day) [42]. A wider range of exposure was noted in Japan (0.008–1.582 µg/kg bw/day, mean 0.215 µg/kg bw/day) [16]; and Taiwan (0–2.67 µg/kg bw/day, median 0.25 µg/kg bw/day) [47].

Direct comparisons with these studies are challenging due to methodological differences. The duplicate diet method used in this study, while limited to 10 days of sample collection, offers a more accurate reflection of population exposure compared to methods relying on food frequency questionnaires or national databases, which often approximate portion sizes and acrylamide levels. Differences may also partly arise from variations in dietary habits between countries and, more significantly, from the differing methodologies employed, which can influence the results. For instance, Vryonidis et al. [48] demonstrated significant discrepancies in acrylamide intake estimates when comparing data derived from hemoglobin adduct levels (0.24 µg/kg bw/day) to those obtained through probabilistic modelling based on dietary surveys (0.26–0.30 µg/kg bw/day). Exposure estimations based on databases and dietary surveys often fail to account for all consumed foods, exact portion sizes, and precise acrylamide levels, relying instead on approximations of average consumption and acrylamide content in a broad range of processed foods. In contrast, although the evaluation period for duplicate diet studies is shorter, these involve the collection of meals over several consecutive days, providing a more accurate and desirable method for describing population exposure.

### 3.5. Limitations and Uncertainties

The estimation of acrylamide exposure through the diet in the context of this research presents various limitations and uncertainties associated with the consumed foods, their acrylamide levels, the study subjects, and the employed methodology, among other factors. This study considers a limited sample size, as exposure was evaluated in two centers over a limited time period, including the assessment of the diet consumed over two weeks. Additionally, only the consumption of food from Monday to Friday was considered, without accounting for weekends, during which food consumption may vary compared to weekdays. The study estimates a scenario in which the canteen attendee consumed the complete menu, without considering variability in consumption among subjects. It also does not consider the possibility that foods outside the canteen menu could be consumed, which might contribute to acrylamide exposure. Another limitation is the assigned weight to each serving, as only one example of each food or dish was weighed.

The acrylamide concentration in each food can be a new source of uncertainty, related to the analytical method and the detection and quantification limits of the determination. Added to this are the values assigned to samples with acrylamide levels below the detection/quantification limit in the upper and lower exposure scenarios. Culinary habits and the foods used for dish preparation can contribute to another source of uncertainty, influenced by the regional or seasonal differences.

Another factor is the body weight of the subjects. When individual body weights are not known, an average body weight value is applied, which can introduce its own uncertainty. Regarding the methodology, other tools for assessing acrylamide exposure, such as the use of biomarkers, could also be employed. However, in that case, it is not possible to discriminate between acrylamide exposure through diet or through other sources, potentially overestimating contaminant exposure compared to the methodology used in the present study.

## 4. Conclusions

This study highlights the importance of assessing acrylamide exposure in institutional diets, particularly among elderly populations who rely on pre-determined menus. The findings indicate that daily dietary acrylamide exposure in senior center canteens ranged from 0.36 to 0.54 µg/kg bw/day, with women generally exhibiting higher exposure levels than men because of lower body weight. The margin of exposure (MOE) for neoplastic effects was as low as 314, significantly below the recommended threshold of 10,000, suggesting a potential health risk. Among the analyzed foods, French fries (27.3 µg/serving) and Spanish omelet (21.6 µg/serving) were the main contributors to acrylamide intake, followed by stews, soups, and creams (ranging from 16.1 to 5.8 µg/serving). Although some of these dishes are often overlooked due to their relatively low acrylamide content, their frequent consumption and large portion sizes can substantially contribute to daily intake.

## Figures and Tables

**Table 1:** (**A**–**D**) Different menus gathered from senior centers.

**(A) Menus from Senior Center 1, Week 1**				
	**Breakfast**	**Lunch**	**Afternoon Snack**	**Dinner**
Monday	Milk Instant decaffeinated coffee Muffin	Stewed beans Breaded dab with French fries Orange Bread	Milk Instant cocoa Biscuits	Cauliflower gratin Breaded pork fillet with ham and cheese Yogurt Whole wheat bread
Tuesday	Milk Instant decaffeinated coffee Toasted bread with oil/butter	Soup Typical chickpea stew made with pork and beef meat and vegetables Banana Bread	Milk Instant cocoa Biscuits	Carrot puree Breaded eggs with bechamel sauce and sautéed vegetables Yogurt Whole wheat bread
Wednesday	Milk Instant decaffeinated coffee Muffin	Breadcrumbs with pork meat and vegetables Salmon stew with vegetables Apple Bread	Milk Instant cocoa Biscuits	Pork and beef meat and vegetable soup Breaded chicken with French fries Yogurt Whole wheat bread
Thursday	Milk Instant decaffeinated coffee Muffin	Noodles with pork meat and vegetables Breaded tilapia with salad Apple Bread	Milk Instant cocoa Biscuits	Vegetable puree Spanish omelet with roasted red peppers Yogurt Whole wheat bread
Friday	Milk Instant decaffeinated coffee Toasted bread with oil/butter	Rice with pork meat and vegetables Chicken and garlic stew with French fries Apple Bread	Milk Instant cocoa Biscuits	Sautéed green beans Assorted cold cuts Yogurt Whole wheat bread
**(B) Menus from Senior Center 1, Week 2**				
	**Breakfast**	**Lunch**	**Afternoon Snack**	**Dinner**
Monday	Milk Instant decaffeinated coffee Muffin	Stewed lentils Breaded ham with bechamel sauce and salad Pear Bread	Milk Instant cocoa Biscuits	Fish soup Spanish omelet with roasted red peppers Yogurt Whole wheat bread
Tuesday	Milk Instant decaffeinated coffee Muffin	Rice with chicken Stewed pork with tomato and vegetables Banana Bread	Milk Instant cocoa Biscuits	Sautéed vegetables Breaded tilapia with French fries Yogurt Whole wheat bread
Wednesday	Milk Instant decaffeinated coffee Toasted bread with oil/butter	Soup Typical chickpea stew made with pork and beef meat and vegetables Pear Bread	Milk Instant cocoa Biscuits	Breaded squid Tomato salad Yogurt Whole wheat bread
Thursday	Milk Instant decaffeinated coffee Muffin	Macaroni with pork meat and tomato Fried eggs with French fries Apple Bread	Milk Instant cocoa Biscuits	Assorted cold cuts Yogurt Whole wheat bread
Friday	Milk Instant decaffeinated coffee Muffin	Stewed beans Breaded hake with sautéed vegetables Banana Bread	Milk Instant cocoa Biscuits	Mackerel with roasted red peppers Yogurt Whole wheat bread
**(C) Menus from Senior Center 2, Week 1**				
	**Breakfast**	**Lunch**	**Afternoon Snack**	**Dinner**
Monday	Fruit juice Milk Instant decaffeinated coffee Mix of cereals and dairy products	Mixed salad Rice with pork meat, fish and vegetables Profiteroles Bread	Milk Instant cocoa Bakery products	Tomato soup Tuna omelet Mandarin oranges Bread
Tuesday	Fruit juice Milk Instant decaffeinated coffee Mix of cereals and dairy products	Stewed beans Tuna patties with salad Pear Bread	Milk Instant cocoa Bakery products	Zucchini puree Fried anchovies with roasted pepper salad Yogurt Bread
Wednesday	Fruit juice Milk Instant decaffeinated coffee Mix of cereals and dairy products	Roasted vegetables Potato stew with beef meat Yogurt Bread	Milk Instant cocoa Bakery products	Rice soup Fresh cheese with quince jam Mandarin orange Bread
Thursday	Fruit juice Milk Instant decaffeinated coffee Mix of cereals and dairy products	Fideuá with seafood Breaded pork fillet with ham and cheese Banana Bread	Milk Instant cocoa Bakery products	Mushroom cream Breaded hake with oven-baked potatoes Roasted apple Bread
Friday	Fruit juice Milk Instant decaffeinated coffee Mix of cereals and dairy products	Seafood soup Fried eggs with sautéed vegetables Custard Bread	Milk Instant cocoa Bakery products	Sautéed red cabbage Zucchini omelet Yogurt Bread
**(D) Menus from Senior Center 2, Week 2**				
	**Breakfast**	**Lunch**	**Afternoon Snack**	**Dinner**
Monday	Fruit juice Milk Instant decaffeinated coffee Mix of cereals and dairy products	Stewed lentils Ham croquettes with salad Pear Bread	Milk Instant cocoa Bakery products	Vegetable soup Breaded cod Apple pie Bread
Tuesday	Fruit juice Milk Instant decaffeinated coffee Mix of cereals and dairy products	Stuffed eggplants with pork meat Potato stew with fish and seafood Yogurt Bread	Milk Instant cocoa Bakery products	Vegetable puree Roasted ham with oven-baked potatoes Mandarin orange Bread
Wednesday	Fruit juice Milk Instant decaffeinated coffee Mix of cereals and dairy products	Roasted red pepper salad Macaroni with tuna Banana Bread	Milk Instant cocoa Bakery products	Pork and beef meat and vegetable soup Vegetable omelet French toast Bread
Thursday	Fruit juice Milk Instant decaffeinated coffee Mix of cereals and dairy products	Soup Typical chickpea stew made with pork and beef meat and vegetables Custard Bread	Milk Instant cocoa Bakery products	Scrambled eggs with mushrooms Baked mako shark with oven-baked potatoes Mandarin orange Bread
Friday	Fruit juice Milk Instant decaffeinated coffee Mix of cereals and dairy products	Mixed salad Rice with pork meat, fish and vegetables Mandarin orange Bread	Milk Instant cocoa Bakery products	Tomato soup Stewed chicken with vegetables and French fries Yogurt Bread

**Table 2:** (**A**,**B**). Acrylamide content (µg/kg), edible serving (g), and acrylamide contribution (µg/serving) in foods/meals compounding menus provided by senior centers.

**(A) Senior Center 1**						
**Meal**	**Foods**	***n***	**FoodEx2 Code**	**Acrylamide Content** **(µg/kg)**	**Edible Serving** **(g)**	**Acrylamide Contribution** **(µg/Serving)**
Breakfast and afternoon snack	Instant decaffeinated coffee	10	A03GR	2831 ± 181	2	5.7 ± 0.4
	Instant cocoa	10	A03HG	261 ± 30	14	3.6 ± 0.4
	Muffins	7	A00BC	21 ± 5	31	0.9 ± 0.1
	Toasted bread	3	A006D	34 ± 7	23	0.8 ± 0.2
	Biscuits	10	A009X	122 ± 120	21	2.7 ± 2.7
Lunches and dinners	Stewed beans	2	A03VT	33 ± 25	413	11.6 ± 5.4
	Stewed lentils	1	A00QD#F28. A07GM	27	282	7.6
	Carrot puree	1	A03XY#F04. A05JG	31	41	12.5
	Pork and beef meat and vegetable soup	1	A041V	22	432	9.4
	Fish soup	1	A041X	40	353	14.2
	Typical chickpea stew made with pork and beef meat and vegetables	2	A03VK#F04. A00SL$F28. A07GM	17 ± 4	339	5.8 ± 0.1
	Breadcrumbs with pork meat and vegetables	1	A007A#F04. A022Y$F04. A00HC$F04. A05CN$F04. A05CP$F28. A07GT	101	156	15.8
	Breaded ham with bechamel sauce	1	A022T#F04. A043X$F28. A07HK	33	117	3.8
	Breaded pork fillet with ham and cheese	1	A03XD	24	86	2.1
	Breaded eggs with bechamel sauce	1	A032B#F04. A043X$F28. A07HK	23	105	2.4
	Breaded chicken	1	A01SP#F28. A07HK$F28. A07GS	26	102	2.6
	Spanish omelet	2	A03YN#F28. A00ZT	161 ± 6	134	21.6 ± 0.1
	Roasted red peppers	2	A05CN#F28. A07GX	160 ± 10	42	6.7 ± 0.3
	French fries	5	A0BYV	213 ± 158	69	13.9 ± 11.9
	Whole wheat bread	10	A005E	29 ± 7	49	1.4 ± 0.5
	Bread	10	A004Y	28 ± 0.6	48	1.3 ± 0.1
**(B) Senior center 2**						
**Meal**	**Foods**	***n***	**FoodEx2 code**	**Acrylamide Content** **(µg/kg)**	**Edible Serving** **(g)**	**Acrylamide Contribution** **(µg/Serving)**
Breakfast and afternoon snack	Instant decaffeinated coffee	10	A03GR	192 ± 4	2	0.4 ± 0.1
	Instant cocoa	10	A03HG	20 ± 2	10	0.2 ± 0.1
	Mix of cereals and dairy products	10	A06DX	45 ± 2	124	5.5 ± 0.2
Lunches and dinners	Stuffed eggplants with pork meat	1	A03YF#F04. A00JD$F04. A049S$F04. A16FE	36	290	10.5
	Stewed beans	1	A03VT	23	405	9.2
	Seafood soup	1	A03XK	29	555	16.1
	Tomato soup	2	A041N	26 ± 15	486	14.0 ± 12.3
	Tuna patties	1	A0FBT#F02. A00CC04. A00ZD	41	104	4.2
	Breaded pork fillet with ham and cheese	1	A03XD	32	101	3.2
	Vegetable omelet	1	A03YQ	65	150	9.8
	Zucchini omelet	1	A03YN#F04. A00JR	55	180	9.9
	Rice with pork meat, fish and vegetables	2	A041D	25 ± 5	335	8.4 ± 2.5
	Sautéed vegetables	1	A03YD	25	309	7.7
	Oven-baked potatoes	3	A011R	20 ± 9	106	2.0 ± 0.6
	French fries	1	A0BYV	304	90	27.3
	Apple pie	1	A00BA#F04. A01DJ	30	93	2.8

The data are presented as mean ± SD. The variable “*n*” represents the number of times the food/meal was collected throughout the study period. Only dishes with acrylamide concentrations > LOQ (15 µg/kg) are shown (47 out of 156 foods/meals analyzed in senior center 1 and 82 out of 148 foods/meals analyzed in senior center 2). SD is not provided when *n* = 1, as only one portion of the food was analyzed. All servings were edible.

**Table 3:** (**A**,**B**). Edible serving (g) and acrylamide contribution (µg/serving) in foods/meals compounding menus provided by the canteens of the senior centers with acrylamide content < LOQ (applied in the upper exposure scenario).

**(A) Senior Center 1**					
**Meal**	**Foods**	***n***	**FoodEx2 Code**	**Edible Serving** **(g)**	**Acrylamide Contribution** **(µg/Serving)**
Lunches and dinners	Noodles with pork meat and vegetables	1	A041V#F04. A007D	527	7.9
	Rice with pork meat and vegetables	1	A041J	305	4.6
	Rice with chicken	1	A041H	362	5.4
	Macaroni with pork meat and tomato	1	A040Q#F04. A049S$F04. A16FE	368	5.5
	Sautéed vegetables	1	A03YD	322	4.8
	Vegetable puree	1	A03XY	367	5.5
	Cauliflower gratin	1	A03YF#F04. A00FR	216	3.2
	Sautéed green beans	1	A00PG#F28. A07GT	186	2.8
	Breaded dab	1	A02AS#F28. A07HK$F28. A07GS	67	1.0
	Salmon stew with vegetables	1	A028P#F04. A044E	137	2.0
	Breaded tilapia	2	A028A#F28. A07HK$F28. A07GS	68	1.0 ± 0.1
	Chicken and garlic stew	1	A01SP#F04. A045A$F28. A07GM	114	1.7
	Stewed pork with tomato and vegetables	1	A03VK	169	2.5
	Fried eggs	1	A032C	45	0.7
	Breaded hake	1	A02CB #F28. A07HK	84	1.3
	Sautéed vegetables	2	A03YD	119	1.8 ± 0.3
	Breaded squid	1	A02JJ#F28. A07HL$F28. A07GV	77	1.2
	Mackerel with roasted red peppers	1	A02CR#F04. A05CNA05CN$F28. A07GX	99	1.5
**(B) Senior center 2**					
**Meal**	**Foods**	***n***	**FoodEx2 code**	**Edible Serving** **(g)**	**Acrylamide Contribution** **(µg/Serving)**
Lunches and dinners	Roasted vegetables	1	A03YF	169	2.5
	Roasted red pepper salad	1	A042D#F04. A05CN	150	2.3
	Potato stew with beef meat	1	A03VJ	360	5.4
	Fideuá with seafood	1	A040Q#F04. A0EYX	229	3.4
	Sautéed red cabbage	1	A00GA#F28. A07GT	179	2.7
	Stewed lentils	1	A00QD#F04. A025C$F28. A07GM	512	7.7
	Potato stew with fish and seafood	1	A03XQ#F04. A0EYX	417	6.3
	Roasted pepper salad	1	A05CN#F28. A07GX	161	2.4
	Zucchini pure	1	A03XY#F04. A00JR	429	6.4
	Mushroom cream	1	A03XY#F04. A00TQ	451	6.8
	Vegetable soup	1	A041S	306	4.6
	Vegetable puree	1	A03XY	392	5.9
	Pork and beef meat and vegetable soup	1	A041V	359	5.4
	Roasted ham	1	A023K	62	0.9
	Fried eggs	1	A032C	122	1.8
	Ham croquettes	1	A022TA022T#F04. A043X$F28. A07HL$F28. A07GS	124	1.9
	Macaroni with tuna	1	A007S#F04. A0FBT	285	4.3
	Typical chickpea stew made with pork and beef meat and vegetables	1	A03VK#F04. A00SL$F28. A07GM	303	4.5
	Tuna omelet	1	A03YN#$F04. A0FBT	85	1.3
	Fried anchovies	1	A02DD#F28. A07GS	41	0.6
	Quince jam	1	A01DR#F28. A07JQ	66	1.0
	Breaded hake	1	A02CB#F28. A07HK$F28. A07GS	91	1.4
	Breaded cod	1	A02BV#F28. A07HK$F28. A07GS	185	2.8
	Scrambled eggs with mushrooms	1	A03YN#$F04. A00TQ	261	3.9
	Baked mako shark	1	A052E#F28. A07GX	145	2.2
	Stewed chicken with vegetables	1	A03VK#F04. A01SP	213	3.2
	Roasted apple	1	A01DJ#F28. A07GX	114	1.7
	Profiteroles	1	A00AK	58	0.9
	French toast	1	A0DRD#F04. A02LT$F04. A 0BY6$F04. A019V$F28. A07GV	132	2.0
	Custard	1	A02PX	120	1.8 ± 0.1
	Bread	20	A004Y	45	0.7 ± 0.1
Afternoon snack	Bakery products	10	A009T	35	0.5 ± 0.1

The acrylamide concentration in all samples was below the LOQ but above the LOD, so each was assigned a value of 15 μg/kg. The variable “*n*” refers to the number of times the food was consumed during the two-week testing period. SD is not provided when *n* = 1, as only one portion of the food was analyzed.

**Table 4:** Minimum and maximum relative contribution of food groups to the total daily acrylamide intake (%) in senior center canteens for both lower and upper exposure scenarios.

**Lower Exposure Scenario**					
	**Coffee** **(%)**	**Cereal-Based Products (%)**	**Potato-Based Dishes** **(%)**	**Cocoa** **(%)**	**Other Foods/Meals** **(%)**
Senior center 1					
Week 1	11.8–17.3	8.0–20.7	0–53.5	7.6–11.1	0–58.0
Week 2	7.9–33.4	8.9–45.7	0–70.6	5.1–21.5	0–46.2
Senior center 2					
Week 1	1.0–6.3	13.9–90.4	0	0.5–3.3	0–84.6
Week 2	1.0–4.4	13.8–63.3	0–68.3	0.5–2.3	0–61.6
Both centers	1.0–33.4	8.0–90.4	0–70.6	0.5–21.5	0–84.6
**Upper Exposure Scenario**					
	**Coffee** **(%)**	**Cereal-Based Products (%)**	**Potato-Based Dishes** **(%)**	**Cocoa** **(%)**	**Other Foods/Meals** **(%)**
Senior center 1					
Week 1	9.2–15.3	7.7–19.3	0–42.7	5.9–9.9	20.1–59.7
Week 2	7.9–23.7	6.8–32.0	0–54.1	5.1–15.3	21.7–46.2
Senior center 2					
Week 1	0.9–2.3	15.4–43.7	0–31.9	0.4–1.2	20.8–83.4
Week 2	0.8–1.7	14.7–32.1	0–54.2	0.4–0.9	30.0–71.2
Both centers	0.8–23.7	6.8–43.7	0–54.2	0.4–15.3	20.1–83.4

**Table 5:** Acrylamide daily exposure from consuming menus served in senior center canteens and assessment of the associated risk.

	Women		Men	
	LB	UB	LB	UB
Senior center 1				
Exposure range (µg/kg bw/day)	0.52 ± 0.23 a	0.62 ± 0.22 a	0.47 ± 0.21 a	0.55 ± 0.19 a
MOE for neurotoxic effects	820	691	923	307
MOE for neoplastic effects	324	273	365	777
Senior center 2				
Mean exposure (µg/kg bw/day)	0.28 ± 0.19 b	0.46 ± 0.15 a	0.25 ± 0.17 b	0.41 ± 0.13 a
MOE for neurotoxic effects	1519	934	1708	1051
MOE for neoplastic effects	600	369	675	415
*p* value	0.021	0.069	0.021	0.069
Both centers				
Mean exposure (µg/kg bw/day)	0.40 ± 0.24	0.54 ± 0.20	0.36 ± 0.21	0.48 ± 0.18
MOE for neurotoxic effects	1065	795	1198	894
MOE for neoplastic effects	421	314	474	353

Lower exposure scenario (LB). Upper exposure scenario (UB). Different letters represent statistically significant differences in exposure between centers for the same scenario (*p* < 0.05). Significant differences were not detected between the exposure of both sexes in any of the larger centers individually or combined (*p* > 0.05). Exposure data are expressed as mean ± SD.

## Data Availability

The original contributions presented in the study are included in the article, further inquiries can be directed to the corresponding author.
